# *KIT* mutation in a naïve succinate dehydrogenase‐deficient gastric GIST

**DOI:** 10.1002/gcc.22768

**Published:** 2019-06-10

**Authors:** Iva Brcic, Karl Kashofer, Daniela Skone, Bernadette Liegl‐Atzwanger

**Affiliations:** ^1^ Diagnostic and Research Institute of Pathology Medical University of Graz Graz Austria; ^2^ Krankenanstalt Rudolfstiftung Institute of Pathology and Microbiology Vienna Austria

**Keywords:** gastrointestinal stromal tumor, KIT gene, mutation, succinate dehydrogenase gene

## Abstract

Up to 85% of gastrointestinal stromal tumors (GIST) harbor mutually exclusive mutations in the *KIT* or the *PDGFRA* gene. Among others, known as wild type GIST, succinate dehydrogenase (SDH)‐deficient tumors develop due to genetic or epigenetic alterations in any of four SDH genes. Herein, we present a unique case of SDH‐deficient GIST with an unusual heterogeneous SDHA and SDHB staining pattern and mutations detected in the *SDHA* and *KIT* gene. A 50‐year‐old patient presented with a 5 cm large gastric tumor with a multinodular/plexiform growth pattern, mixed epithelioid and spindle cell morphology, and focal pronounced nuclear atypia with hyperchromasia and high mitotic activity. Immunohistochemically, CD117 and DOG‐1 were positive. SDHB and SDHA stains showed loss of expression in some of the nodules, whereas others presented with an unusually weak patchy positivity. Molecular analysis revealed a point mutation in exon 5 of the *SDHA* gene and a mutation in exon 11 of the *KIT* gene. We hypothesize that based on the allele frequency of *SDHA* and *KIT* mutations the tumor is best regarded as SDH‐deficient GIST in which the *SDHA* mutation represents the most likely driver mutation. The identified *KIT* mutation raises the distinct possibility that the *KIT* mutation is a secondary event reflecting clonal evolution. This is the first case of a treatment naïve GIST harboring a somatic *SDHA* and a *KIT* mutation, challenging the dogma that oncogenic mutations in treatment naïve GIST are mutually exclusive.

## 1 INTRODUCTION

Gastrointestinal stromal tumor (GIST) is the most common mesenchymal neoplasm of the gastrointestinal tract with an annual incidence of approximately 10‐15 cases per million.^1^ It usually presents sporadically in older adults (median age 60‐65 years) with no gender predilection. GISTs occur throughout the gastrointestinal tract, but most commonly affect the stomach and the small intestine. Up to 85% of GISTs harbor mutually exclusive mutations in *KIT* or *PDGFRA*.^2^, ^3^ These mutations are responsible for upregulation of crucial signaling pathways including MAPK and PI3K‐AKT. Most *KIT/PDGFRA* mutated GISTs respond to the RTK inhibitor imatinib; however, treatment response is mainly depending on tumor genotype.^4^, ^5^

Tumors devoid of *KIT* and *PDGFRA* mutations are known as RTK‐wild type (WT) GISTs. Over the last years, it became apparent that the so‐called “WT‐GIST group” is quite heterogeneous with regards to clinical phenotype and molecular characteristics.^6^ Based on recent advances in molecular pathology, GISTs can be sub‐classified in an SDH‐competent and an SDH‐deficient group, irrespective of whether they are sporadic or familial/genetic.

SDH is an enzyme complex located in the inner mitochondrial membrane and is composed of four subunits (SDHA‐D) mapping to 5p15.33, 1p36.13, 1q23.3, and 11q23.1, respectively. The SDH complex connects the oxidation of succinate to fumarate in the Krebs cycle to the reduction of coenzyme Q in the mitochondrial electron transport chain.^7^ Genetic or epigenetic alterations of any of the four SDH genes cause destabilization of the SDH‐complex and result in accumulation of succinate and activation of cellular pathways leading to increased angiogenesis and cellular proliferation.^7^ Destabilization of any of the SDH subunits can be demonstrated by immunohistochemistry, based on the loss of SDHB expression.^7^, ^8^, ^9^

The SDH‐competent GISTs include tumors with KIT, PDGFRA, NF1, and BRAF mutations as well as tumors with rare described mutations in *ARID1A, ARID1B, CBL, FGFR1, NRAS, HRAS, KRAS, MAX*, *MEN1,* and *PIK3CA* and novel gene fusions, like *KIT‐PDGFRA* and *ETV6‐NTRK3*.^10^, ^11^, ^12^

In contrast, the SDH‐deficient GIST group includes the majority of pediatric GISTs, some sporadic adult/young adult cases, and rare syndromic GISTs developing in association with the Carney‐Stratakis‐Syndrome and the Carney triad. The underlining genetic cause for the complete loss or substantial reduction in SDHB protein expression by immunohistochemistry is heterogeneous including germline and somatic mutations, promotor hypermethylation, and deletions. Also, SDH‐deficient GISTs are characterized by distinctive multinodular/plexiform architecture, epithelioid or mixed morphology, common lymph node metastasis, and indolent behavior of metastases.^6^, ^8^, ^9^, ^13^

In Carney‐Stratakis syndrome, SDH deficiency is caused by germline mutations in *SDHB*, *SDHC,* or *SDHD*.^10^, ^14^, ^15^ However, the Carney triad and other pediatric cases are most commonly caused by epigenetic silencing of the *SDHC* gene through promoter hypermethylation.^10^, ^16^ Very recently, Benn et al. found that pathogenic *SDHA‐C* variants present as germline events in the general population with tumors not driven by these mutations in up to 25.6%.^17^ The authors used a Bayesian approach to calculate penetrance for *SDHA* variants at 1.7% (95% CI 0.8% to 3.8%). Furthermore, *SDHA* mutations have been demonstrated in “apparently” sporadic adult gastric RTK‐WT GISTs.^18^, ^19^, ^20^ Analysis of large GIST sample collectives proposed that SDH deficiency is mutually exclusive to KIT/PDGFRA/BRAF/NF1/KRAS mutations.^8^, ^21^

Herein, we report a case of imatinib naïve SDHB‐deficient GIST with an unusual immunohistochemical expression profile for SDHA and SDHB and unique molecular findings.

## 2 PATIENT AND METHODS

### 2.1 Immunohistochemical analysis

Four micrometers thick formalin‐fixed, paraffin‐embedded whole‐tissue sections were analyzed. Immunohistochemistry was performed on a Dako autostainer with the detection Kit Dako REAL Envision Plus, K5007, using a rabbit anti‐CD117 polyclonal antibody (c‐kit; clone A4502, 1:1000 dilution; Dako, Glostrup, Denmark), a rabbit anti‐CD117 monoclonal antibody DOG1 (Clone: SP31, 1:100 dilution, Thermo Fischer, Waltham, MA), a mouse anti‐SDHA monoclonal antibody (clone 2E3GC12FB2AE2, 1:750, Abcam, Cambridge, MA), and a mouse anti‐SDHB monoclonal antibody (clone 21A11AE7, 1:1000 dilution, Abcam, Cambridge, MA).

### 2.2 Molecular analysis

#### 2.2.1 DNA workflow

Genomic DNA was extracted from formalin‐fixed, paraffin‐embedded (FFPE) material (5‐8 unstained, 10 μm thick sections). H&E stained sections of FFPE Blocks were examined, and areas of high tumor content (tumor sample) or without any tumor content (corresponding normal sample) were marked on the slide. Up to 10 consecutive 10 μm thick sections were used for microdissection with a needle to enrich for tumor or normal cell content. DNA was isolated using the Maxwell RSC DNA FFPE kit according to manufacturer's instructions. DNA was quantified by picogreen fluorescence. Twenty nanograms DNA were used for multiplex PCR reactions using a custom Ion Torrent AmpliSeq panel covering selected genes of interest.

#### 2.2.2 Targeted next‐generation sequencing

Mutational analysis was performed using next‐generation sequencing (NGS) (Ion AmpliSeq technology MUG GIST Panel ‐ searching for mutations in the *KIT, PDGFRA, PDGFRB, K‐RAS, N‐RAS, H‐RAS, BRAF, SDHA, SDHB, SDHC, SDHD, NF1, CDKN2A, TP53,* and *RB1* gene) in the Laboratory for Diagnostic Genome Analysis, Institute of Pathology, Medical University of Graz, Austria. After PCR amplification, adapter ligation and purification were performed by the Ion Torrent Ampliseq Library Kit 2.0. NGS libraries were sequenced on Ion Torrent Proton using the Ion PI Hi‐Q Sequencing 200 kit. Reads were aligned to the human reference genome (hg19), and variants were called using Torrent Variant Caller v5.6. Variants were visually inspected to remove artefacts and subsequently annotated with open source software (ANNOVAR and SNPeff). All analyses were carried out as technical duplicates with two separate NGS libraries generated for each DNA sample.

#### 2.2.3 Sanger sequencing

Fifty nanograms of DNA were used for PCR amplification using primers KIT Ex11 fwd 5′‐TGTTCTCTCTCCAGAGTGCTCTAAT‐3′ and KIT Ex11 rev 5′‐AAACAAAGGAAGCCACTGGA‐3′. Amplified PCR products were subjected to Sanger sequencing reaction using BigDye terminator v1.1 Kit (Thermo Fischer, Waltham, MA). Sequences were analyzed using SeqMan Pro Software (DNASTAR) and the 3500 Genetic Analyser (Thermo Fisher).

### 2.3 Patient

**Figure 1:**
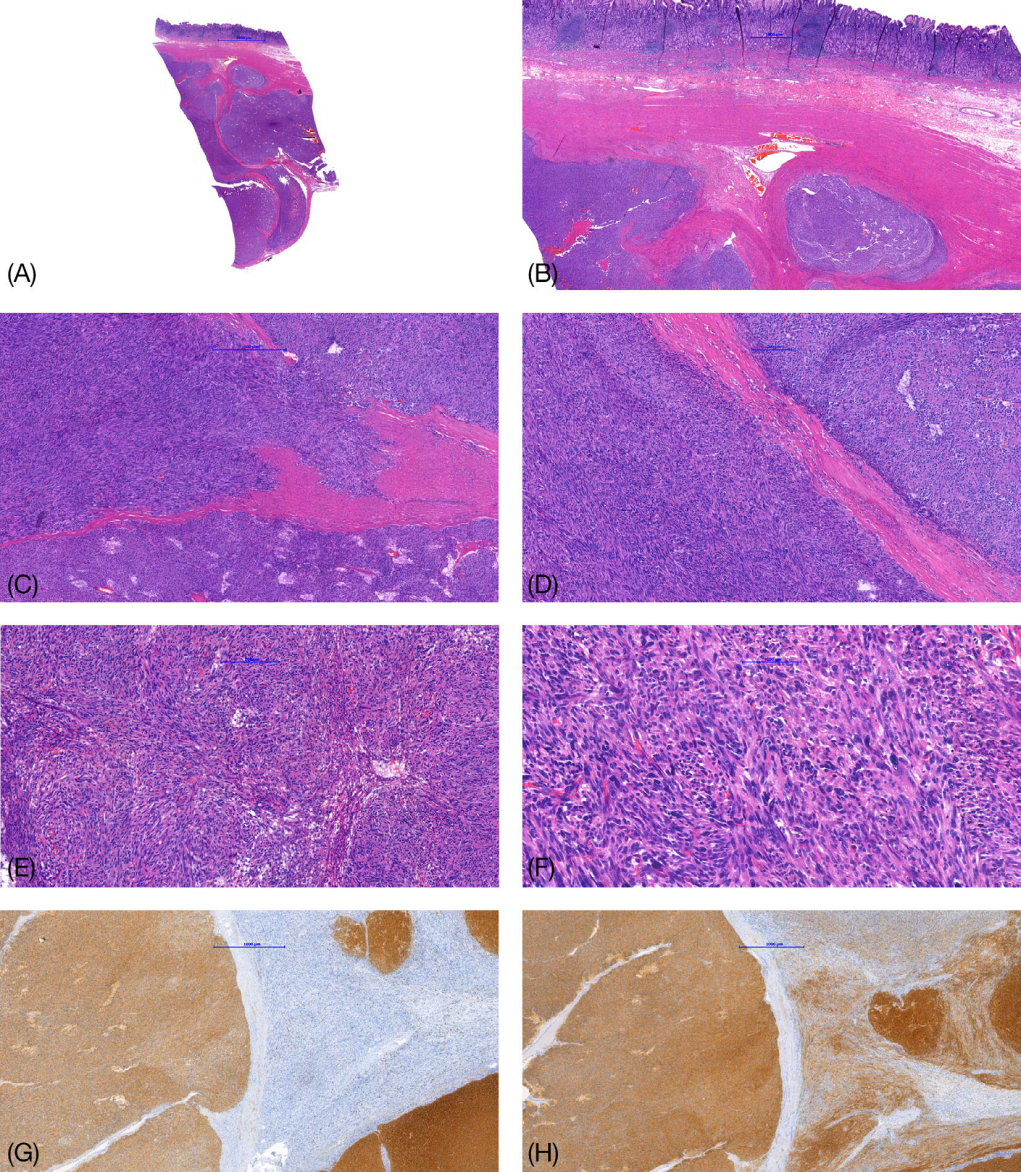
Histological findings. The gastric gastrointestinal stromal tumor with coarsely multilobulated/plexiform growth pattern (A and B). The tumor is composed of spindle or epithelioid cells grouped in short fascicles and whorls with foci of pronounced nuclear atypia with hyperchromasia (C and D). Higher power of spindle and epitheliod area (E) and of area with obvious atypia (F). Immunhistochemically, the tumor shows positivity for DOG‐1 and CD117 (G and H) [Color figure can be viewed at wileyonlinelibrary.com]

**Figure 2:**
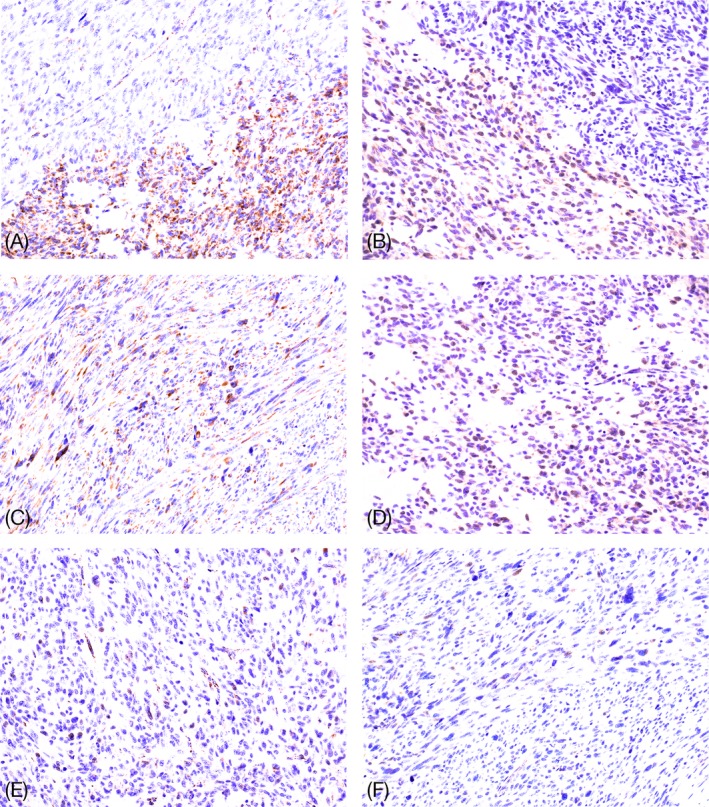
Findings of SDHA (A, C, E) and SDHB (B, D, F) immunostains. (A and B) The expression is lost in one part of the tumor, and retained in other showing (especially with SDHB) an unusual weak patchy positivity. Higher power of positive (C and D) and negative areas (E and F). Cytoplasmic stain of the endothelial cells is used as the positive internal control [Color figure can be viewed at wileyonlinelibrary.com]

A 50‐year‐old patient presented with recurrent abdominal pain. Medical history and physical examination were unremarkable. Laboratory findings showed no significant alterations except for a moderate, normocytic anemia (hemoglobin 9.5 g/L). Gastroscopy revealed a partially ulcerated submucosal lesion in the antrum. A biopsy specimen showed ulcerated antral mucosa, tumor tissue was not present. The patient underwent a distal gastrectomy for complete resection. Gross examination of the specimen revealed a 5 cm, well‐demarcated intramural mass located in the gastric antrum. On cut surface, the tumor was multinodular, solid, fleshy, tan white in appearance with small cysts and areas of hemorrhage. On histology, the tumor showed a typical multinodular/plexiform growth pattern and was composed of spindle cells grouped in short fascicles and whorls as well as sheets of epithelioid cells (Figure 1A,B). A pale eosinophilic cytoplasm with syncytial cell borders surrounded the tumor cells. In addition, microcystic stromal change and foci of pronounced nuclear atypia with hyperchromasia as well as high mitotic activity (15 mitoses per 5 mm^2^) were present (Figure 1C‐F). Immunohistochemistry showed a multifocal strong expression of CD117 (C‐KIT) and DOG‐1 (Figure 1G,H). SDHB and SDHA stains showed loss of expression in some of the nodules, whereas others presented with an unusually weak patchy positivity (Figure 2A‐F).

**Figure 3:**
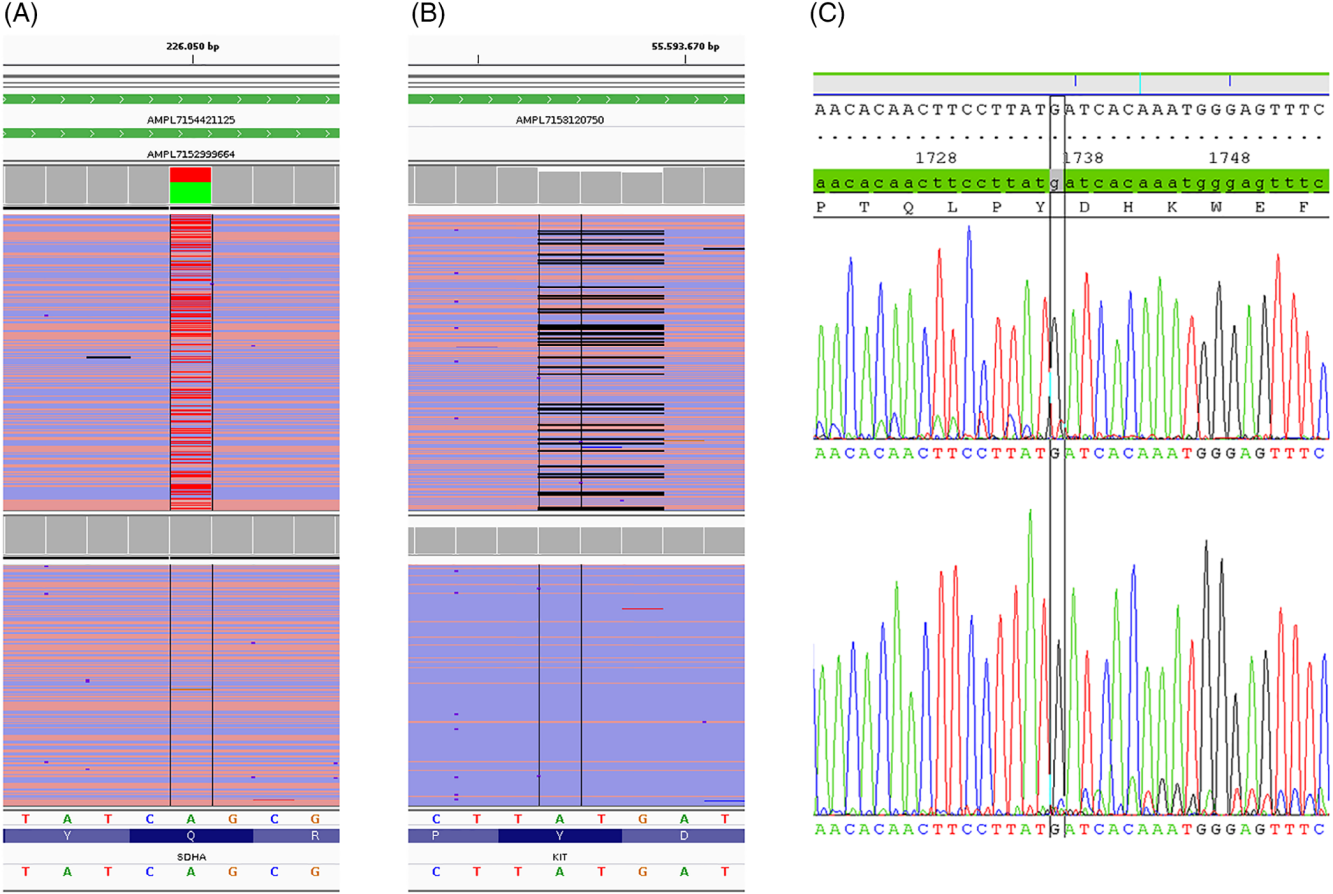
SDHA Q170L and KIT D579del detection. The SDHA Q170L mutation is clearly visible in NGS reads from tumor tissue (A, top) and absent in reads from corresponding normal tissue (A, bottom). The KIT D579del is clearly visible in NGS reads from tumor tissue (B, top) and absent in reads from corresponding normal tissue (B, bottom). Confirmation of KIT D579del by Sanger Sequencing in reverse (C, top) and forward (C, bottom) direction. Black vertical bars correspond to mutation/deletion site [Color figure can be viewed at wileyonlinelibrary.com]

Molecular analysis using NGS revealed a point mutation in exon 5 of the *SDHA* (p.Q170L) gene. The minor allele frequency (MAF) of the mutation was 43%, (1707 mut, 2283 wt reads) and 41% (1650 mut and 2344 wt reads) in duplicate analyses. In addition, a mutation in exon 11 of the *KIT* (p.D579del) gene (MAF 13%, 530 mut, 3434 wt reads and MAF 12%, 458 mut and 3514 wt reads in duplicate analyses) was found (Figure 3). We did not detect any additional somatic mutations within the genomic regions covered by the NGS panel. Sanger sequencing confirmed results obtained by NGS. As there have been reports of germline mutations in *SDH* genes we additionally analyzed non‐tumoral tissue micro‐dissected from FFPE material of resection margins without tumor cells. *KIT* and *SDHA* mutations were not present in normal tissue (no mutated reads in SDHA Q170 locus (>3990 and >3984 wt reads) or KIT D579 locus (> 3973 and >3971 wt reads).

## 3 DISCUSSION

Up to 85% of GISTs harbor mutually exclusive mutations in the *KIT* or *PDGFRA* gene.^2^, ^3^ Based on the underlining genotype, the majority of these tumors respond to treatment with imatinib or second‐line treatment with sunitinib or regorafenib.^4^, ^5^, ^22^, ^23^ However, almost half of RTK‐WT GISTs demonstrate deficiency of the tumor suppressor complex SDH as a distinct alternative mechanism of oncogenesis.^8^, ^18^, ^20^

SDH‐deficient tumors develop due to genetic or epigenetic alterations in any of four SDH genes: *SDHA*, *SDHB*, *SDHC,* or *SDHD* (or collectively *SDHx*) and respond poorly to standard targeted therapy. Within the SDH‐deficient GIST group, distinctive subgroups based on molecular and genetic aspects of the defect were identified.^10^ This group includes patients with the Carney‐Stratakis syndrome (gastric GIST and paragangliomas) carrying germline mutations in *SDHB‐D,* a subgroup of sporadic young adult cases with *SDHA* mutations as well as patients with epigenetic silencing of the *SDHC* gene mainly reported in syndromic Carney triad (gastric GIST, paragangliomas, pulmonary chondromas, and other tumors).^10^, ^15^, ^16^, ^17^

Independently of the underlining molecular mechanism, SDH‐deficient GISTs show unique clinical, pathological, and molecular features distinctive from SDH‐competent GIST.^6^ They usually occur in the stomach in young female patients, are often multifocal, show a distinctive multinodular/plexiform growth pattern, an epithelioid or mixed epithelioid/spindle cell morphology, and tend to metastasize to lymph nodes and liver. Metastases in this setting may be commonly associated with an indolent clinical course.^6^

Large studies analyzing the SDH status using immunohistochemistry for the SDHB subunit supported the notion that SDH deficiency is in general mutually exclusive to other known oncogenic mechanisms.^7^, ^8^, ^9^ The most extensive series investigated 756 gastric GIST by SDHB immunohistochemistry and identified 66 *SDHB* deficient GIST, whereas all 378 non‐gastric GISTs were found to be *SDHB*‐competent.^6^ Heterogeneity of the staining pattern for SDHB was not reported, and staining was performed on a tissue microarray where individual cores might not represent the full morphologic spectrum of the tumors. Molecular testing of the 66 *SDHB* deficient gastric GISTs revealed no mutations in *KIT, PDGFRA, BRAF,* or *SDH* genes. However, *SDHA* mutational analysis was not included, and only a limited number of exons in *SDHB‐D* were covered. In 2013, different groups demonstrated that loss of SDHA protein expression by immunohistochemistry reliably predicts the presence of *SDHA* mutations in GIST and can, therefore, be used to select patients with *SDH*‐deficient GIST for further molecular analysis.^19^, ^20^

In the English literature, only single‐case reports exist demonstrating an *SDH*‐deficient GIST with an RTK mutation.^24^ This finding, however, is mainly associated with germline *SDH* mutations. However, a very well‐documented recent case reported by Belinsky et al. described oncogenic somatic mutations in *PDGFRA* and *SDHB* in a metastatic GIST after treatment with several RTKs.^25^

In contrast, we describe a unique case of a treatment naïve GIST of the stomach with typical multinodular/plexiform growth pattern, mixed epithelioid and spindle cell morphology, microcystic stromal changes, foci of pronounced nuclear atypia with hyperchromasia, and high mitotic activity (15 mitoses per 5 mm^2^). Interestingly immunohistochemistry showed a typical multifocal strong expression of CD117 (C‐KIT) and DOG‐1; however, an unusual heterogeneous SDHA and SDHB immunohistochemical staining pattern (depending on the blocks used for IHC) was observed. Molecular analysis, confirmed by two independent methods, revealed a point mutation in exon 5 of the *SDHA* (p.Q170L) gene (MAF 43%) and a mutation in exon 11 of the *KIT* (p.D579del) gene (MAF 15%). Given recently shown high incidence and low penetrance of *SDHA* variants,^17^ we performed NGS analysis of non‐tumor tissue of the patient to exclude the possibility of these mutations being already present in the germline of the patient. This concurrent analysis of non‐tumoral tissue revealed the homozygous wild type reference sequence in both genetic loci and thus clearly confirming the somatic nature of both reported mutations.

Based on the allele frequency of *SDHA* and *KIT* mutations, our tumor is best defined as *SDH*‐deficient GIST in which SDH loss is most likely the oncogenic driver. Furthermore, the identified convincing *KIT* mutation in a small allele fraction raises the distinct possibility that the *KIT* mutation is a second event reflecting a clonal evolution. Although there is the dogma that oncogenic mutations in GIST are mutually exclusive, there is good evidence that there are well‐documented exceptions to this rule.

## 4 CONCLUSION

To the best of our knowledge, this is the first case of a treatment naïve GIST harboring a somatic *SDHA* mutation best regarded as a potential driver mutation in addition to a somatic *KIT* mutation explained as a second event reflecting a clonal evolution. This case, together with another recently reported case with *SDHB* and *PDGFRA* D842V mutations, challenges the dogma that oncogenic mutations in GIST are mutually exclusive. Expanded molecular testing in the era of NGS may be of diagnostic and therapeutic value and may be the rational for including patients into treatment trials based on the molecular landscape of tumors.
